# Trends in emergency department visits related to acute alcohol consumption before and during the COVID-19 pandemic in the United States, 2018–2020

**DOI:** 10.1016/j.dadr.2022.100049

**Published:** 2022-03-27

**Authors:** Marissa B. Esser, Nimi Idaikkadar, Aaron Kite-Powell, Craig Thomas, Kurt J. Greenlund

**Affiliations:** a Division of Population Health, National Center for Chronic Disease Prevention and Health Promotion, Centers for Disease Control and Prevention, 4770 Buford Hwy NE, MS-S107-6, Atlanta, GA, 30341, USA; b Division of Injury Prevention, National Center for Injury Prevention and Control, Centers for Disease Control and Prevention, 4770 Buford Hwy NE, MS-S106-8, Atlanta, GA, 30341, USA; c Division of Health Informatics and Surveillance, National Center for Surveillance, Epidemiology, and Laboratory Services, Centers for Disease Control and Prevention, 2500 Century Blvd NE, MS-V25-3, Atlanta, GA, 30345, USA

**Keywords:** Alcohol use, Alcohol-related injury, Alcohol policy, Emergency department surveillance

## Abstract

**Background:**

Excessive drinking accounts for more than 95,000 deaths annually in the United States. Coronavirus disease 2019 (COVID-19) pandemic-related factors (e.g., social, economic, policy) may have affected alcohol consumption. Emergency department (ED) visits involving acute alcohol consumption (referred to as “alcohol-related”) are a useful indicator for assessing changes in alcohol-related harms.

**Methods:**

The 2018–2020 National Syndromic Surveillance Program data, which include nonfatal ED visits from facilities in 49 states and Washington, DC, were analyzed. Trends in the number of alcohol-related ED visits among people ≥15 years, and weekly alcohol-related ED visit rates (per 10,000 total visits) overall, by demographic characteristics, and quarter (Q) were assessed. Quarterly rates for 2018 and 2019 were averaged to increase baseline data stability.

**Results:**

Alcohol-related visits accounted for 1.6% of 60,474,770 total visits (2018), 1.7% of 61,564,380 total visits (2019), and 1.8% of 52,174,507 total visits (2020). The number of alcohol-related ED visits generally increased during the first eight months of 2018 and 2019. However, it sharply declined at the onset of the COVID-19 pandemic in mid-March–mid-April 2020, before resuming pre-pandemic patterns. Alcohol-related ED visits per 10,000 were higher during quarters in 2020 than corresponding quarters in 2018–2019 (Q1: +7.3%, Q2: +23.8%, Q3: +9.7%, Q4: +6.5%).

**Conclusions:**

Alcohol-related ED visit rates per 10,000 total visits increased during 2020 versus 2018–2019, with the greatest relative difference in the second quarter. Fewer people sought ED care in 2020 than 2018–2019 but alcohol-related visits declined to a lesser extent than total visits.

## Highlights

• COVID-19 pandemic-related factors may have affected alcohol use and related harms.
• We analyzed data from emergency department visits in 49 states and Washington, DC.
• Alcohol-related emergency department visit rates increased in 2020 versus 2018–19.
• Alcohol-related ED visit quarterly rates were 7–24% higher in 2020 than 2018–19.
• Population-level alcohol strategies are needed to reduce alcohol-related ED visits.

## 1 Introduction

Excessive alcohol use is responsible for more than 95,000 deaths each year in the US, shortening the lives of those who die by an average of 29 years (Esser et al., 2020). About half of these deaths are associated with the effects of acute drinking (e.g., alcohol poisoning, injuries) (Esser et al., 2020).

Some evidence suggests alcohol consumption may have increased during the COVID-19 pandemic. For example, a small survey of US adults showed a greater prevalence of binge drinking in April compared with February 2020, and found that respondents consumed a greater number of drinks per day (Barbosa et al., 2020). Fluctuations in alcohol consumption may reflect drinking alcohol as a coping mechanism for COVID-19 pandemic-related stress or emotions (Czeisler et al., 2020a), or changes in alcohol availability policies that have occurred in many states since the COVID-19 pandemic began (e.g., expansion of home delivery from online alcohol sales, carryout alcohol, or temporary closures of bars) (National Institute on Alcohol Abuse and Alcoholism, 2021).

ED visits involving acute drinking during the COVID-19 pandemic have not been examined in the US. We assessed trends in ED visits associated with acute alcohol consumption (referred to as “alcohol-related ED visits”) before and during the COVID-19 pandemic in 2018–2020, using data on nonfatal ED visits from the Centers for Disease Control and Prevention's (CDC) National Syndromic Surveillance Program (NSSP).

## 2 Methods

### 2.1 Data source

The NSSP evolved from BioSense, which originally launched to provide surveillance for potential bioterrorism-related illnesses. The NSSP now facilitates monitoring changes over time for various public health problems (e.g., substance use (Nolan et al., 2017) or drug overdose (Holland et al., 2021)). The NSSP receives deidentified ED visits from hospitals in 49 states (excluding Hawaii) and Washington, DC, with approximately 71% of all US EDs participating (Centers for Disease Control and Prevention, 2021). Data in this study include December 31, 2017 through January 2, 2021 to align with the surveillance weeks to capture each day of 2018 and 2020. This activity was reviewed by the CDC and was conducted consistent with applicable federal law and CDC policy (see, e.g., 45 CFR part 46; 21 CFR part 56; 42 USC §241(d), 5 USC §552a, 44 USC §3501 et seq).

### 2.2 Query of ED visits related to acute alcohol consumption

A query was designed for monitoring ED visits associated with acute alcohol consumption based on discharge diagnosis codes and chief complaint text (see Supplemental Tables 1 and 2). The query includes International Classification of Diseases, Tenth Revision, Clinical Modification (ICD)−10-CM diagnosis codes with evidence of acute alcohol use. This query does not include codes that only indicate chronic alcohol use (e.g., alcoholic liver cirrhosis) without an indication of acute drinking.

### 2.3 Analyses

We assessed trends in the number of alcohol-related ED visits among people aged ≥15 years and weekly alcohol-related ED visit rates (per 10,000 ED visits) overall, by population characteristics, and by quarter (Q) during 2020 compared with corresponding quarters during 2018–2019. Because of fluctuations in facilities reporting to the NSSP over time, data were included from the facilities that were consistently reporting throughout the study period so that only facilities with more complete data were included (Radhakrishnan et al., 2022) (where the facility-level average percentages for reporting diagnosis codes to the NSSP was ≥70%). Analyses focused on rates per 10,000 ED visits because of the substantial decline in total ED visits during the onset of the COVID-19 pandemic (Adjemian et al., 2021; Hartnett et al., 2020).

We calculated weekly rates and then calculated relative percent differences and standard deviations in quarterly rates, based on average weekly rates, of alcohol-related ED visits per 10,000 visits during 2020 compared with corresponding quarters during 2018–2019. We averaged the rates for corresponding quarters in 2018 and 2019 to increase the stability of the baseline data for comparisons with 2020 data. Analyses were conducted using RStudio version 1.3.1093–1.

## 3 Results

In this analysis of the NSSP data, there were 988,205 alcohol-related ED visits in 2018 (1.6% of 60,474,770 total visits), 1026,874 in 2019 (1.7% of 61,564,380 total visits), and 953,667 in 2020 (1.8% of 52,174,507 total visits).

### 3.1 Trends in the number of alcohol-related ED visits

**Fig. 1:**
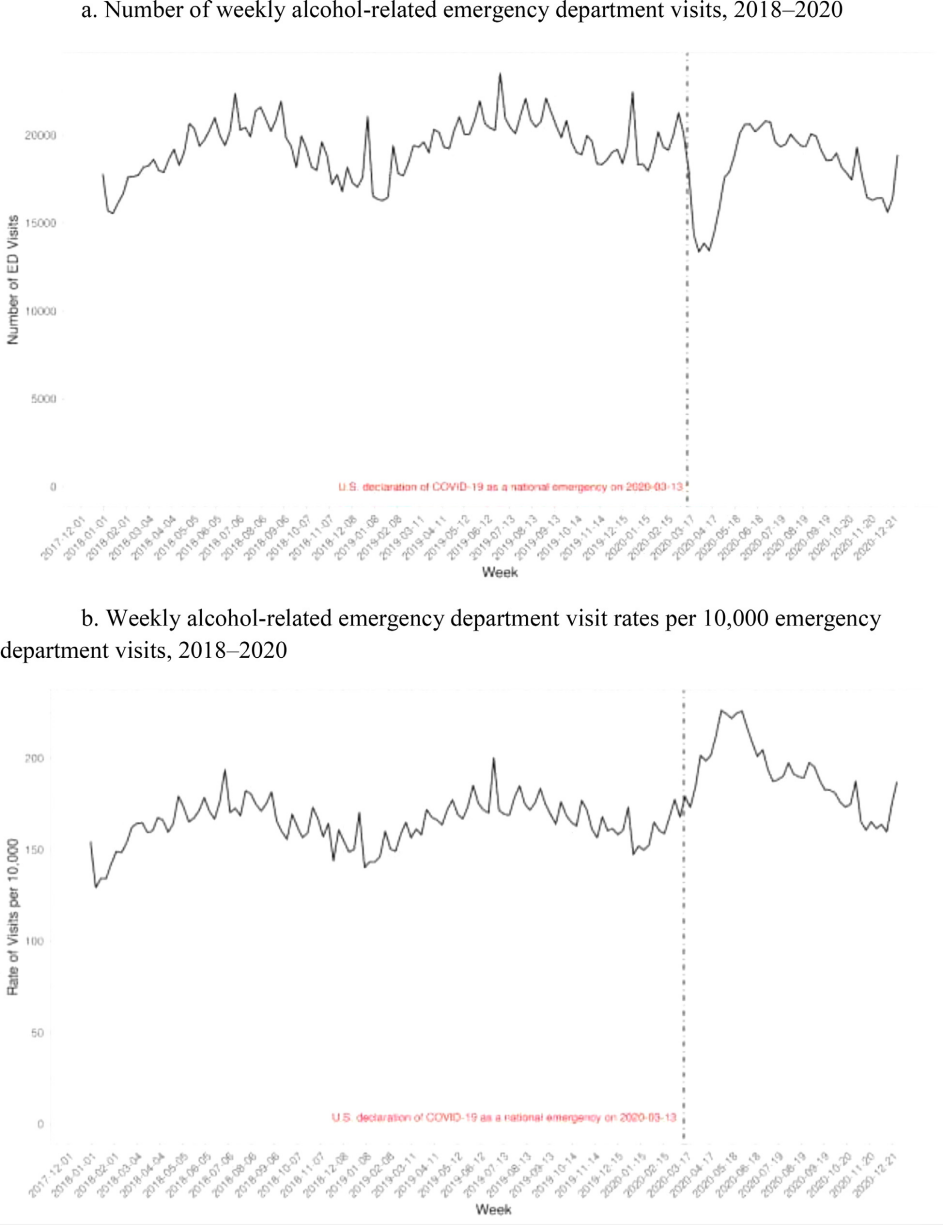
a. Number of weekly alcohol-related emergency department visits, 2018–2020. Fig. 1b. Weekly alcohol-related emergency department visit rates per 10,000 emergency department visits, 2018–2020.

The number of alcohol-related ED visits generally increased for the first eight months of 2018 and 2019. In 2020, the number sharply declined at the onset of the COVID-19 pandemic in mid-March–mid-April, coinciding with the US declaration of the COVID-19 national emergency on March 13, 2020 (Fig. 1A). The number of alcohol-related ED visits then sharply increased from mid-April to late-June, and then returned to levels similar to corresponding weeks in 2018 and 2019, before generally declining slowly until mid-December 2020, and increasing at the end of the year.

### 3.2 Trends in rates of alcohol-related ED visits

The average of weekly rates of alcohol-related ED visits per 10,000 total visits increased from 163.4 in 2018 and 166.7 in 2019 to 184.8 in 2020. In 2020, rates of alcohol-related ED visits per 10,000 total visits increased consistently from late-January to a peak in late-April through early-June, more sharply than in 2018 or 2019, and the peak rates were higher than any rate during 2018–2019 (Fig. 1B). Alcohol-related ED visit rates then declined until mid-December 2020, where rates were comparable to corresponding weekly rates during 2019. Towards late-December, alcohol-related ED visit rates increased and were higher in 2020 than 2018 or 2019.

Average quarterly alcohol-related ED visit rates per 10,000 visits were higher during 2020 than corresponding quarters in 2018–2019. Rates were 7.3% higher in Q1, 23.8% higher in Q2, 9.7% higher in Q3, and 6.5% higher in Q4 (Table 1). For both males and females, alcohol-related ED visit rates were higher in each quarter of 2020 than corresponding 2018–2019 quarters. Rates ranged from 3.5% (Q4)–16.7% (Q2) higher among males and from 6.0% (Q4)–25.0% (Q2) higher among females. Among adults aged ≥25 years, in each age group, rates were higher in all quarters of 2020 than 2018–2019. Among people aged 15–24 years, only the Q1 and Q2 rates were higher in 2020 than 2018–2019. By geographic region and within the 10 HHS regions, changes in alcohol-related ED visit rates during 2020 versus 2018–2019 varied by location.

**Table 1:** Alcohol-related emergency department visit rates^a^ per 10,000 emergency department visits and relative difference^b^ in alcohol-related emergency department visit rates by quarter and by characteristics, 2018–2019 and 2020.

	Quarter 1, January–March			Quarter 2, April–June			Quarter 3, July–September			Quarter 4, October–December		
**Characteristic**	Rate, 2018– 2019	Rate, 2020	Relative difference % (SD)	Rate, 2018–2019	Rate, 2020	Relative difference % (SD)	Rate, 2018–2019	Rate, 2020	Relative difference % (SD)	Rate, 2018–2019	Rate, 2020	Relative difference % (SD)
**Overall**	152.3	163.4	7.3 (9.3)	170.6	211.3	23.8 (8.3)	174.4	191.3	9.7 (8.2)	161.2	171.6	6.5 (8.8)
**Sex**												
Males	248.4	262.3	5.6 (5.5)	276.4	322.6	16.7 (4.9)	279.2	298.7	7.0 (4.9)	259.8	269.0	3.5 (5.3)
Females	80.6	86.0	6.6 (17.0)	90.0	112.6	25.0 (15.2)	93.4	101.5	8.6 (14.6)	86.5	91.8	6.0 (15.8)
**Age group (years)**												
15–24	110.8	113.1	2.1 (15.9)	116.1	130.2	12.1 (15.2)	128.1	120.9	−5.6 (13.8)	124.0	120.2	−3.1 (14.2)
25–34	158.8	164.4	3.5 (11.1)	174.2	211.8	21.6 (10.1)	177.5	192.8	8.6 (9.9)	169.0	179.1	6.0 (10.4)
35–44	199.9	217.4	8.8 (8.8)	221.0	281.1	27.2 (8.0)	222.1	257.6	16.0 (7.9)	214.0	238.0	11.2 (8.2)
45–64	232.2	248.4	7.0 (7.6)	264.8	322.3	21.7 (6.7)	265.0	290.7	9.7 (6.7)	241.2	255.0	5.7 (7.3)
≥65	50.6	61.4	21.5 (34.9)	59.1	80.4	36.1 (29.9)	62.3	74.1	19.1 (28.4)	56.6	64.2	13.5 (31.2)
**Geographic regions**^c^												
Northeast	184.8	199.2	7.8 (8.5)	205.8	241.4	17.3 (7.6)	209.9	225.7	7.5 (7.5)	196.0	201.8	3.0 (8.0)
Southeast	112.1	126.5	12.9 (14.0)	126.8	173.5	36.9 (12.3)	131.0	151.4	15.6 (11.9)	118.9	142.1	19.6 (13.2)
South Central	119.4	119.9	0.4 (13.1)	128.4	150.6	17.3 (12.2)	129.1	131.9	2.2 (12.1)	118.0	121.7	3.2 (13.3)
Midwest	142.6	150.0	5.2 (11.0)	161.9	196.8	21.5 (9.7)	166.4	181.9	9.3 (9.4)	153.3	154.8	1.0 (10.2)
West	201.8	211.5	4.8 (7.8)	223.4	282.7	26.6 (7.0)	224.9	252.1	12.1 (7.0)	212.1	225.5	6.3 (7.4)
**HHS Region**^d^**(Regional office)**												
Region 1 (Boston, MA)	235.7	256.3	8.7 (9.1)	257.4	329.5	28.0 (8.3)	258.6	296.1	14.5 (8.3)	246.3	257.1	4.4 (8.7)
Region 2 (New York City, NY)	227.3	242.5	6.7 (9.4)	259.6	299.8	15.5 (8.3)	257.4	271.1	5.4 (8.3)	237.7	246.6	3.8 (9.0)
Region 3 (Washington DC)	118.9	129.3	8.7 (18.0)	129.6	147.6	14.0 (16.5)	138.2	146.0	5.6 (15.5)	129.3	131.6	1.8 (16.6)
Region 4 (Atlanta, GA)	112.1	126.5	12.9 (19.1)	126.8	173.5	36.9 (16.9)	131.0	151.4	15.6 (16.4)	118.9	142.1	19.6 (18.0)
Region 5 (Chicago, IL)	142.6	150.0	5.2 (15.0)	161.9	196.8	21.5 (13.2)	166.4	181.9	9.3 (12.9)	153.3	154.8	1.0 (14.0)
Region 6 (Dallas, TX)	119.4	119.9	0.4 (17.9)	128.4	150.6	17.3 (16.7)	129.1	131.9	2.2 (16.6)	118.0	121.7	3.2 (18.2)
Region 7 (Kansas City, MO)	134.3	140.8	4.9 (16.0)	150.1	184.2	22.7 (14.3)	148.4	153.4	3.4 (14.4)	139.9	135.9	−2.8 (15.3)
Region 8 (Denver, CO)	311.7	330.7	6.1 (6.9)	343.7	442.5	28.7 (6.2)	338.7	398.9	17.8 (6.3)	322.4	357.4	10.9 (6.6)
Region 9 (San Francisco, CA)	157.5	162.6	3.2 (13.6)	179.0	222.0	24.0 (12.0)	183.5	195.1	6.3 (11.7)	166.9	171.6	2.8 (12.8)
Region 10 (Seattle, WA)	199.3	205.0	2.9 (10.8)	211.8	256.9	21.3 (10.1)	211.5	231.6	9.5 (10.1)	206.8	214.4	3.7 (10.4)

SD: standard deviation.

a Quarterly rates are calculated based on weekly average rates.

b Relative differences in alcohol-related emergency department visit rates per 10,000 were calculated based on the rates for each quarter during 2020 compared with corresponding quarters during 2018–2019.

c States are categorized into one of the geographic regions based on the 10 regions of the Department of Health and Human Services (HHS). The *Northeast* region includes HHS Region 1 (Connecticut, Maine, Massachusetts, New Hampshire, Rhode Island, and Vermont), HHS Region 2 (New Jersey and New York), and HHS Region 3 (Delaware, District of Columbia, Maryland, Pennsylvania, Virginia, and West Virginia); the *Southeast* region includes HHS Region 4 (Alabama, Florida, Georgia, Kentucky, Mississippi, North Carolina, South Carolina, and Tennessee); the *South Central* region includes HHS Region 6 (Arkansas, Louisiana, New Mexico, Oklahoma, and Texas); the *Midwest* region includes HHS Region 5 (Illinois, Indiana, Michigan, Minnesota, Ohio, and Wisconsin) and HHS Region 7 (Iowa, Kansas, Missouri, and Nebraska); and the *West* region includes HHS Region 8 (Colorado, Montana, North Dakota, South Dakota, Utah, and Wyoming), HHS Region 9 (Arizona, California, and Nevada), and HHS Region 10 (Alaska, Idaho, Oregon, and Washington). Data are not available for one state (Hawaii).

d States are categorized in one of the 10 regions of the Department of Health and Human Services (HHS) including Region 1: Connecticut, Maine, Massachusetts, New Hampshire, Rhode Island, and Vermont; Region 2: New Jersey and New York; Region 3: Delaware, District of Columbia, Maryland, Pennsylvania, Virginia, and West Virginia; Region 4: Alabama, Florida, Georgia, Kentucky, Mississippi, North Carolina, South Carolina, and Tennessee; Region 5: Illinois, Indiana, Michigan, Minnesota, Ohio, and Wisconsin; Region 6: Arkansas, Louisiana, New Mexico, Oklahoma, and Texas; Region 7: Iowa, Kansas, Missouri, and Nebraska; Region 8: Colorado, Montana, North Dakota, South Dakota, Utah, and Wyoming; Region 9: Arizona, California, and Nevada; Region 10: Alaska, Idaho, Oregon, and Washington. Data are not available for one state (Hawaii).

Trends in the number and rates of alcohol-related ED visits by population groups are shown in the online supplement. Alcohol-related ED visit rates among both males and females were generally higher than during 2020 than 2018–2019. By age group, alcohol-related ED visit rates during 2020 were higher, on average, than 2018–2019. However, among people aged 15–24 years, rates were slightly lower in the second half of 2020 than 2018–2019. By geographic region and HHS region, alcohol-related ED visit rates peaked during mid-April to mid-June, and the rates remained somewhat elevated but followed more typical seasonal fluctuations during the second half of 2020.

## 4 Discussion

The study assessed more than 174 million total ED visits from facilities that contributed consistently to the NSSP during 2018–2020. We observed that the overall average rate of alcohol-related ED visits per 10,000 total visits increased to 184.8 in 2020, up from 163.4 in 2018 and 166.7 in 2019. The quarterly alcohol-related ED visit rates per 10,000 total visits were 7–24% higher during quarters in 2020 than corresponding quarters in 2018–2019. The greatest relative difference in alcohol-related ED visit rates occurred during the second quarter, which is consistent with another study that showed increases in drinking frequency during May–June 2020 relative to 2019 (Pollard et al., 2020). However, the number of alcohol-related ED visits declined at the beginning of the pandemic, unlike corresponding dates in earlier years. This decline is consistent with the substantial decreases in total ED visits and ED visits for life threatening conditions and coincides with when stay-at-home orders went into effect (Hartnett et al., 2020; Lange et al., 2020). Taken together, these study findings suggest that ED visits for alcohol-related emergencies declined to a lesser extent than ED visits for other causes.

People may have tried to avoid EDs, unless their condition was severe enough to warrant emergency medical attention, including complications associated with acute alcohol consumption, as well as mental health, drug overdose, and violence related outcomes (Holland et al., 2021). Changes in the trends of alcohol-related ED visits during the COVID-19 pandemic may be related to people drinking to cope with social isolation and other stressors such as increases in unemployment (Czeisler et al., 2020a; Wardell et al., 2020).

Several other factors might have influenced alcohol-related ED visit rates in 2020, including changes in the availability of and access to alcohol during the COVID-19 pandemic. The initial closure of bars and restaurants may have temporarily reduced access to alcohol for consumption on-premises, but many states deemed establishments that sold alcohol for consumption off-premises (e.g., liquor stores) as essential businesses, permitting them to remain open (National Institute on Alcohol Abuse and Alcoholism, 2021). In addition, many states implemented legislation that may have increased access to alcohol for off-premise consumption, including allowing direct to consumer shipping and home delivery of alcohol (National Institute on Alcohol Abuse and Alcoholism, 2021). Several states have converted new alcohol carryout and delivery policies into permanent laws (National Institute on Alcohol Abuse and Alcoholism, 2021), increasing the availability of and access to alcohol compared to pre-pandemic times.

This study has limitations. First, the NSSP data are not weighted to represent the US population so the findings may not be generalizable to EDs that do not participate in the NSSP and the use of alcohol-related diagnosis codes may not be uniform across EDs. Second, changes in the demographic characteristics of the patients who visited the participating EDs over time for all causes could also affect the rates reported in this study, as it was not possible to assess patient characteristics such as race and ethnicity or income level. Third, these findings are likely conservative estimates of alcohol-related emergency events compared to non-pandemic times because avoidance of medical care has been reported during the pandemic (Czeisler et al., 2020b). Fourth, these data may underestimate alcohol-related ED visits because discharge diagnoses or chief compliant text relating to alcohol use may be incomplete (Uong et al., 2022). Lastly, it is not possible to attribute changes in alcohol-related ED visits to the pandemic; we were not able to control for factors such as changes in alcohol availability, ED capacity for patients with COVID-19, and the implementation and enforcement of COVID-19 community mitigation measures.

Nevertheless, the general patterns of the rates of alcohol-related ED visits in this study are consistent with the epidemiology of alcohol-attributable deaths (Esser et al., 2020), suggesting the usefulness of this surveillance to inform the prevention of adverse alcohol-related outcomes. In addition, per capita alcohol sales data are consistent with the region-specific study findings on alcohol-related visit rates (e.g., highest in the West and Northeast, and lowest in the South) (Slater and Alpert, 2019).

## 5 Conclusions

Alcohol-related ED visit rates per 10,000 total visits increased during 2020 versus 2018–2019, with the greatest relative difference in the second quarter. While the number of all-cause ED visits declined substantially during the early stage of the COVID-19 pandemic and remained lower during 2020 than 2019 (Adjemian et al., 2021), the number of alcohol-related ED visits did not decrease as much as total ED visits. Strong alcohol policies, including those that increase the price and reduce the availability of and access to alcohol (e.g., increasing alcohol taxes, regulating alcohol outlet density), are associated with decreased excessive drinking and related harms (Community Guide, 2016). A comprehensive approach including more widespread implementation of these effective population-level alcohol policies (Community Guide, 2016), messaging to promote help-seeking for behavioral or mental health support (Holland et al., 2021), and alcohol screening and brief intervention (face-to-face or via electronic modes for providing telehealth services) could help to reduce excessive drinking and alcohol-related ED visits (O'Connor et al., 2018), and the burden of alcohol use on US healthcare systems.

## Contributors' approval statement

All authors have read and approved the final manuscript.

## Contributors

M. Esser, K. Greenlund, and C. Thomas conceptualized the study. M. Esser, N. Idaikkadar and A. Kite-Powell designed the study. N. Idaikkadar and A. Kite-Powell completed the statistical analysis. M. Esser and K. Greenlund drafted the article. M. Esser oversaw the study. All authors interpreted the data, provided substantial intellectual contributions, and reviewed the article.

## Declaration of Competing Interest

No conflict declared.

## Appendix Supplementary materials

Image, application 1
