# Supplementary material for: Trends in emergency department visits related to acute alcohol consumption before and during the COVID-19 pandemic in the United States, 2018–2020
Source: Drug Alcohol Depend Rep. 2022 Mar 27;3:100049. doi: 10.1016/j.dadr.2022.100049 (PMC8957715; doi:10.1016/j.dadr.2022.100049)
Supplement: Supplementary file 1 [file mmc1.docx]

**Supplement**

Supplemental Table 1. Included and excluded discharge diagnosis codes and chief complaint text for query on emergency department visits related to acute alcohol consumption

Supplemental Table 2. Query for emergency department visits related to acute alcohol consumption

Supplemental Figure 1. Number of weekly alcohol-related emergency department visits by sex, 2018–2020

Supplemental Figure 2. Weekly alcohol-related emergency department visit rates per 10,000 emergency department visits by sex, 2018–2020

Supplemental Figure 3. Number of weekly alcohol-related emergency department visits by age group, 2018–2020

Supplemental Figure 4. Weekly alcohol-related emergency department visit rates per 10,000 emergency department visits by age group, 2018–2020

Supplemental Figure 5. Number of weekly alcohol-related emergency department visits by geographic region, 2018–2020

Supplemental Figure 6. Weekly alcohol-related emergency department visit rates per 10,000 emergency department visits by geographic region, 2018–2020

Supplemental Figure 7. Number of weekly alcohol-related emergency department visits by the 10 regions of the Department of Health and Human Services, 2018–2020

Supplemental Figure 8. Weekly alcohol-related emergency department visit rates per 10,000 emergency department visits by HHS region, 2018–2020

**Supplemental** **Table 1. Included and excluded discharge diagnosis codes and chief complaint text for query on emergency department visits related to acute alcohol consumption**

| **Discharge diagnoses and chief compliant** | **Codes and specific terms** |
| --- | --- |
| **Included discharge diagnoses** |  |
| ICD-10-CM elevated blood alcohol level | Y90, R78.0 |
| ICD-10-CM alcohol use; abuse/dependence with intoxication | F10.12, F10.22, F10.9 |
| ICD-10-CM toxic effect of ethanol/unspecified alcohol | T51.0, T51.9 |
| ICD-9-CM idiosyncratic alcohol intoxication | 291.4 |
| ICD-9-CM toxic effect of ethanol/unspecified alcohol | 980.0, 980.9 |
| ICD-9-CM alcohol poisoning | E860.0, E860.1, E860.8, E860.9 |
| ICD-9-CM elevated blood-alcohol level | 790.3 |
| ICD-9-CM alcohol use/abuse with intoxication | 303.0, 305.0 |
| SNOMED alcohol intoxication | 25702006, 191802004, 191804003, 21000000 |
| **Included chief complaints^a^** |  |
| Alcohol | etoh (eoth), alc, pint, pints, drunk, drinks too much, drank too much |
| Alcohol types, brands, and alcoholic beverages | consum, drank, or drink  WITH  beer, wine, tequila, bourb, whiske, vodka, moonshin, tallboys, liquor, 4 loko (4 loco, four loko), bud light, redds, miller light (miller lite), heineken (heinkein), michelob, dos equis, bacardi, coors light, hard cider, jose cuervo, blue moon, twisted tea, crown royal, white claw, jim beam (jim bean), jack dan, wild turkey, margarita, old fashion, mojito, moscow mule, martini, white russian, daiquiri, bloody mary, malt beverage, malt liq, mix drink, mixed drink, scotch, smirnov (smirnoff), hard lemon, sky blue, rum, gin, brandy, captain morgan, hennessy (various misspellings), binge, heavily, heavy |
| **Excluded** **discharge diagnoses** |  |
| ICD-10-CM hepatic fibrosis | K74.0 |
| ICD-10-CM cirrhosis of liver | K74.60, K74.69 |
| ICD-10-CM encounter for blood-alcohol test | Z02.1, Z02.83 |
| ICD-9-CM cirrhosis of liver | 571.5 |
| SNOMED patient encounter | 305058001 |
| **Excluded** **chief complaints** |  |
| Non-alcohol beverages | Drinks too much, drank too much  WITH  water, milk, coke, pepsi, soda, windex, cola, diet,  juice, fluid |
| Not alcohol use | Denies alcohol, not drinking, not eating, not eatting, not eating or drinking, withdraw, delirium, tremens, drug alcohol assessment, drug alcohol test, alcohol blood drug test, drug alcohol screening, quit AND drink, has not drank, denied alcohol, denies any alcohol, denied any alcohol, denies drug alcohol, denied drug alcohol, denies drugs alcohol, denied drugs alcohol, denies drug or alcohol, denied drug or alcohol, denies drugs or alcohol, denied drugs or alcohol, denies any drug alcohol, denied any drug alcohol, denies any drugs alcohol, denied any drugs alcohol, denies any drug or alcohol, denied any drug or alcohol, denies any drugs or alcohol, denied any drugs or alcohol, denies drank, denies drink, denies drunk, denied drank, denied drink, denied drunk |

^a^ Misspellings were included in the text searches (e.g., “eatting”, “jim bean”), in addition to the correct spellings for certain search terms to capture common spelling errors.

**Supplemental** **Table 2. Description of query for emergency department visits related to acute alcohol consumption**

| **Description:**  Using the Electronic Surveillance System for the Early Notification of Community-based Epidemics (ESSENCE) to access the NSSP data, a query was designed for monitoring ED visits associated with acute alcohol consumption based on discharge diagnosis codes and chief complaint text (see Supplemental Table 1). The query includes International Classification of Diseases, Tenth Revision, Clinical Modification (ICD)-10-CM diagnosis codes with evidence of acute alcohol use determined by blood alcohol level, acute alcohol use, alcohol abuse with intoxication, alcohol dependence with intoxication, and the toxic effect of ethanol or unspecified alcohol. Corresponding ICD-9-CM and Systematized Nomenclature of Medicine codes are also included, as those are used in some facilities. In addition, alcoholic beverage types and leading specific alcoholic beverages and brands are included in the query of chief complaint text. Discharge diagnosis codes and chief complaint free text suggesting non-alcoholic beverage or rubbing alcohol consumption, or alcohol withdrawal or detoxification were excluded. |
| --- |
| **Query text:**  ^[;/ ]Y90^,or,^[;/ ]F10.[12]2^,or,^[;/ ]F10.9^,or,^[;/ ]F10[12]2^,or,^[;/ ]F109^,or,^[;/ ]T51.[09]^,or,^[;/ ]T51[09]^,or,^[;/ ]R78.0^,or,^[;/ ]R780^,or,^[;/ ]291.4^,or,^[;/ ]2914^,or,^[;/ ]980.[09]^,or,^[;/ ]E860.[0189]^,or,^[;/ ]790.3^,or,^[;/ ]305.0^,or,^[;/ ]303.0^,or,^[;/ ]3050^,or,^[;/ ]3030^,or,^[;/ ]980[09]^,or,^[;/ ]E860[0189]^,or,^[;/ ]7903^,or,^[;/ ]25702006^,or,^[;/ ]191802004^,or,^[;/ ]191804003^,or,^[;/ ]21000000^,or,(,^etoh^,or,^eoth^,OR,^ alc^,or,alc^,or,^ pint ^,or,^ pints ^,or,^drunk^,OR,(,(,^drinks too much^,or,^drank too much^,),andnot,(,^water^,OR,^milk^,OR,^coke^,OR,^pepsi^,OR,^soda^,or,^windex^,or,^cola^,or,^diet^,or,^juice^,or,^fluid^,),),or,(,(,^consum^,or,^drank^,or,^drink^,),and,(,^beer^,or,^wine^,or,^tequila^,or,^bourb^,or,^whiske^,or,^vodka^,or,^moonshin^,or,^tallboys^,or,^liquor^,or,^4 loko^,or,^4 loco^,or,^four loko^,or,^bud light^,or,^redds^,or,^miller light^,or,^miller lite^,or,^heinkein^,or,^heineken^,or,^michelob^,or,^dos equis^,or,^bacardi^,or,^coors light^,or,^hard cider^,or,^jose cuervo^,or,^blue moon^,or,^twisted tea^,or,^crown royal^,or,^white claw^,or,^jim beam^,or,^jim bean^,or,^jack dan^,or,^wild turkey^,or,^margarita^,or,^old fashion^,or,^mojito^,or,^moscow mule^,or,^martini^,or,^white russian^,or,^daiquiri^,or,^bloody mary^,or,^malt beverage^,or,^malt liq^,or,^mix drink^,or,^mixed drink^,or,^scotch^,or,^smirnov^,or,^smirnoff^,or,^hard lemon^,or,^sky blue^,or,^ rum ^,or,^ gin ^,or,gin ^,or,rum ^,or,^ gin,or,^ rum,or,^brandy^,or,^captain morgan^,or,^ hennes[sye]^,or,^ henes[sye]^,or,hennes[sye]^,or,henes[sye]^,or,^ binge^,or,^heavily^,or,^heavy^,),),),ANDNOT,(,^water^,OR,^milk^,OR,^coke^,OR,^pepsi^,OR,^soda^,or,^windex^,or,^cola^,or,^diet^,or,^juice^,or,^fluid^,or,^denies alcohol^,or,^not drinking^,or,^not eating^,or,^not eatting^,or,^not eating or drinking^,or,^withdraw^,or,^delirium^,or,^tremens^,or,^drug alcohol assessment^,or,^drug alcohol test^,or,^alcohol blood drug test^,or,^drug alcohol screening^,or,^[;/ ]K74.0^,or,^[;/ ]K740^,or,^[;/ ]K74.6[09]^,or,^[;/ ]K746[09],or,^[;/ ]571.5^,or,^[;/ ]5715^,or,^[;/ ]z02.83^,or,^[;/ ]z0283^,or,^without mention of alc^,or,^detox^,or,^[;/ ]305058001^,or,^ rub^,or,^[;/ ]z02.1^,or,^[;/ ]z021^,or,(,^ quit^,and,^ drink^,),or,^has not drank^,or,^denie[sd] alcohol^,or,^denie[sd] any alcohol^,or,^denie[sd] drug alcohol^,or,^denie[sd] drugs alcohol^,or,^denie[sd] drug or alcohol^,or,^denie[sd] drugs or alcohol^, or,^denie[sd] any drug alcohol^,or,^denie[sd] any drugs alcohol^,or,^denie[sd] any drug or alcohol^,or,^denie[sd] any drugs or alcohol^,or,^denie[ds] dr[aui]nk^,) |

**Supplemental** **Figure 1. Number of weekly alcohol-related emergency department visits by sex, 2018–2020**


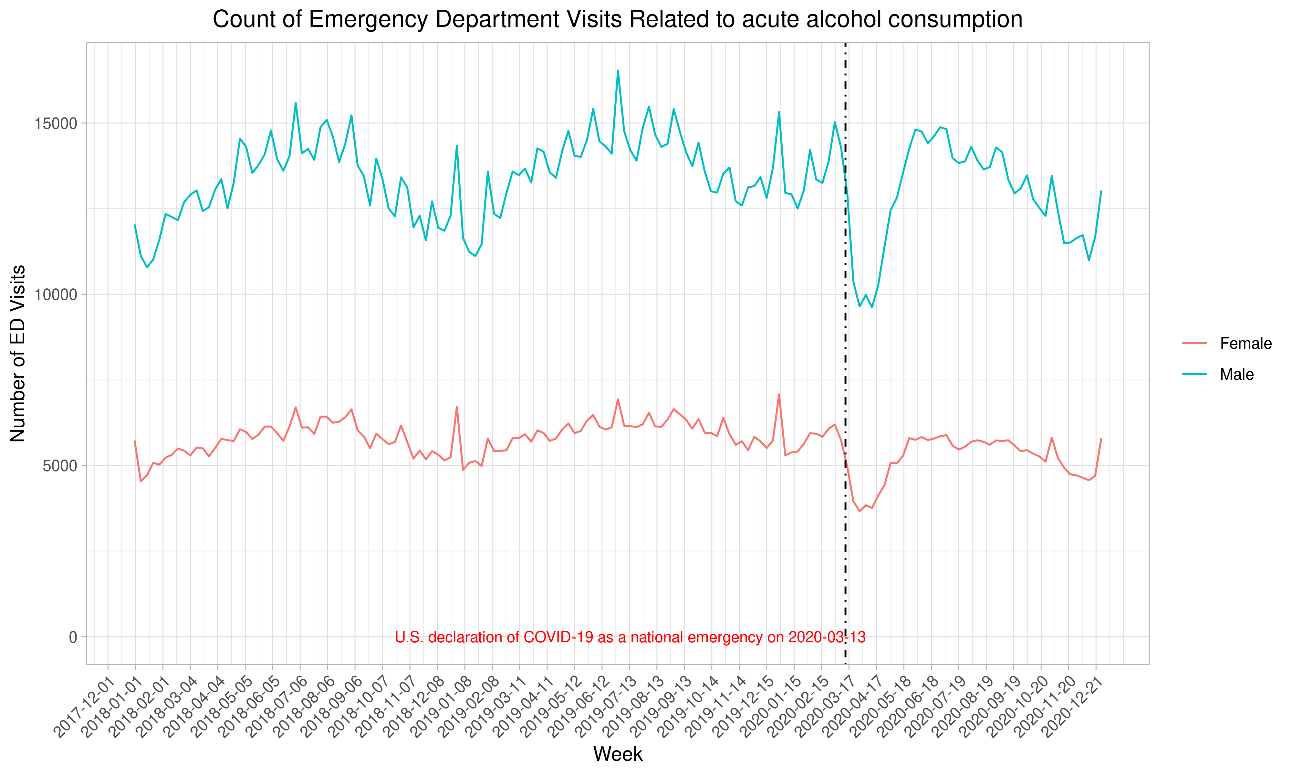


**Supplemental** **Figure 2.** **Weekly alcohol-related emergency department visit rates per 10,000 emergency department visits by sex, 2018–2020**


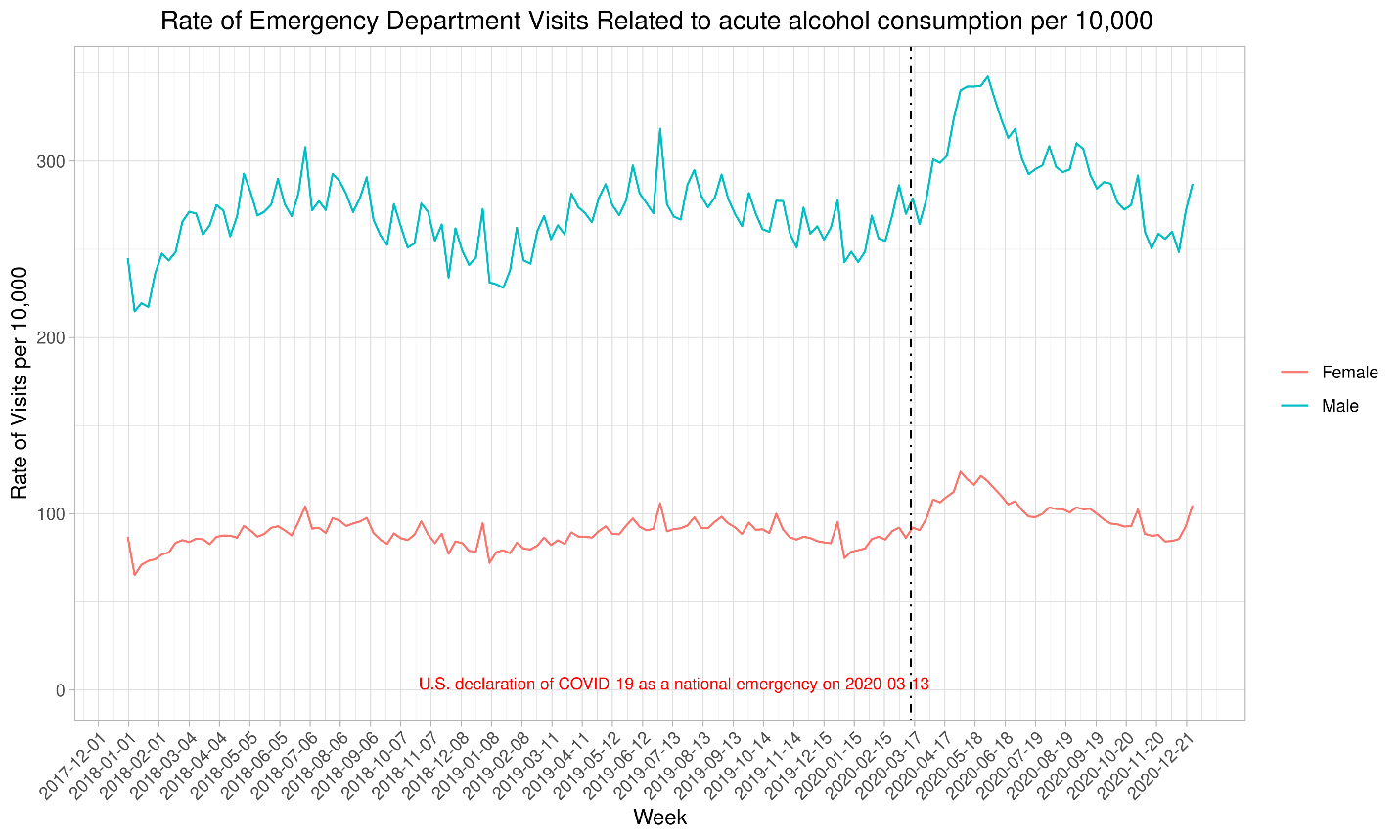


**Supplemental** **Figure 3. Number of weekly alcohol-related emergency department visits by age group, 2018–2020**


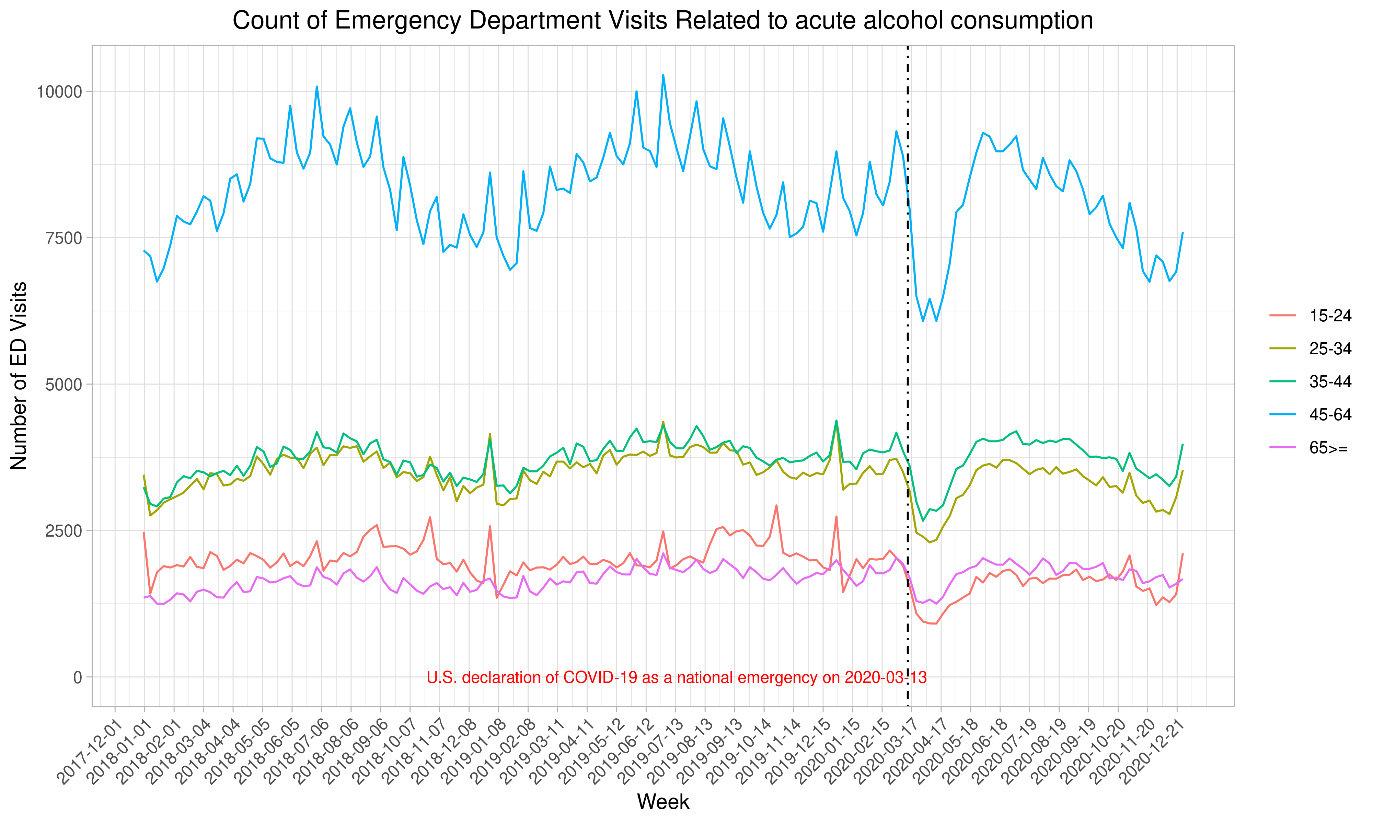


**Supplemental Figure 4. Weekly alcohol-related emergency department visit rates per 10,000 emergency department visits by age group, 2018–2020**


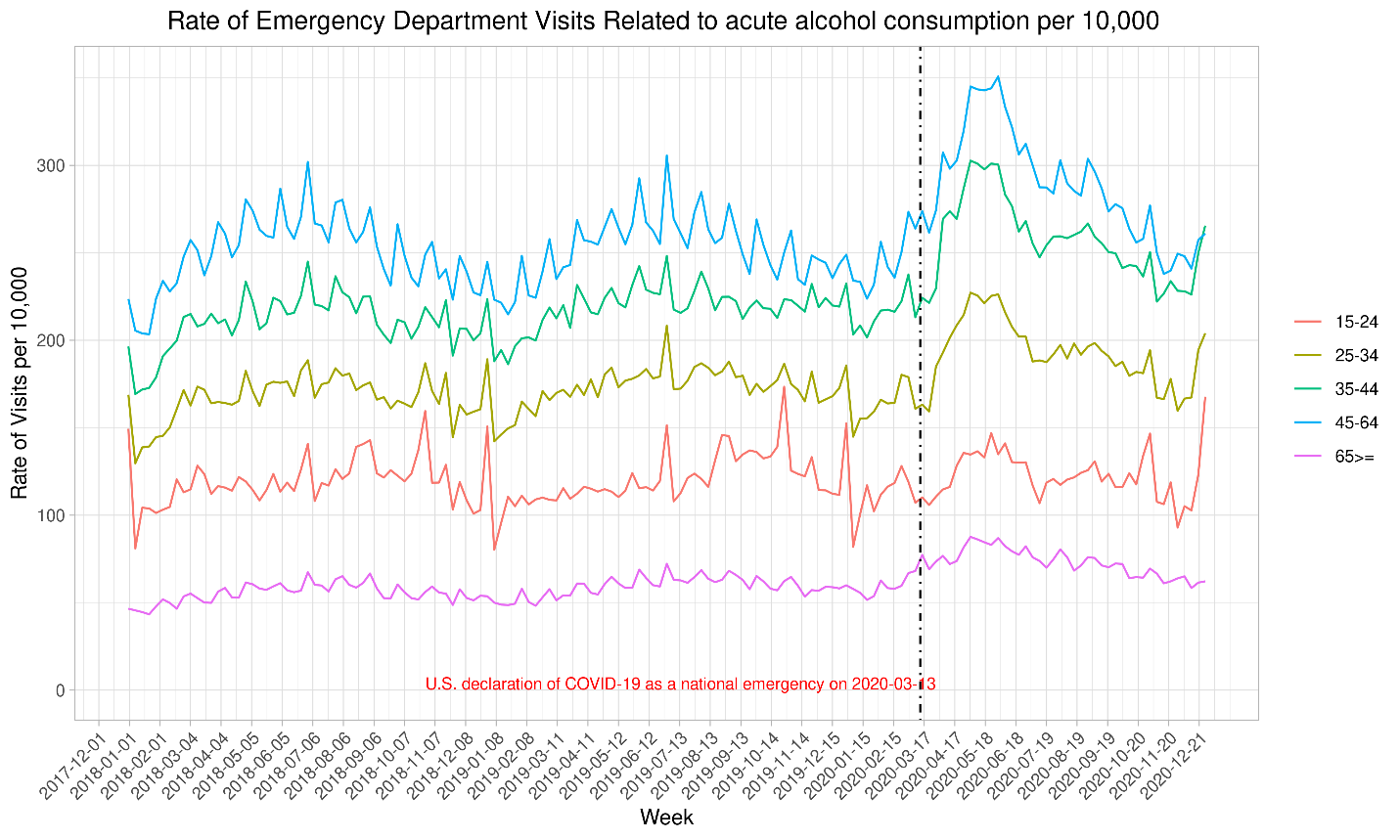


**Supplemental** **Figure 5. Number of weekly alcohol-related emergency department visits by geographic region,^a^ 2018–2020**


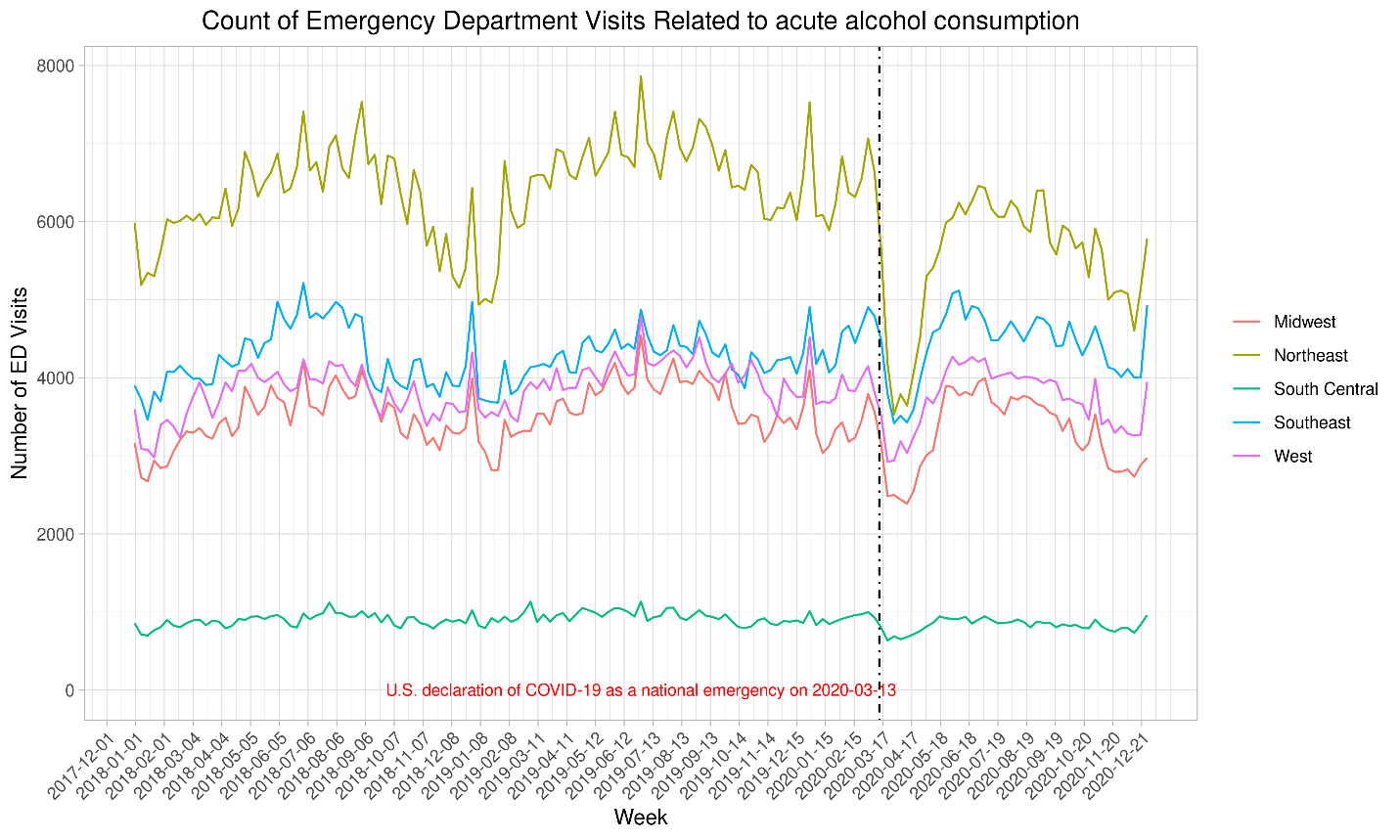


**^a^** States are categorized into one of the geographic regions based on the 10 regions of the Department of Health and Human Services (HHS). The *Northeast* region includes HHS Region 1 (Connecticut, Maine, Massachusetts, New Hampshire, Rhode Island, and Vermont), HHS Region 2 (New Jersey and New York), and HHS Region 3 (Delaware, District of Columbia, Maryland, Pennsylvania, Virginia, and West Virginia); the *Southeast* region includes HHS Region 4 (Alabama, Florida, Georgia, Kentucky, Mississippi, North Carolina, South Carolina, and Tennessee); the *South Central* region includes HHS Region 6 (Arkansas, Louisiana, New Mexico, Oklahoma, and Texas); the *Midwest* region includes HHS Region 5 (Illinois, Indiana, Michigan, Minnesota, Ohio, and Wisconsin) and HHS Region 7 (Iowa, Kansas, Missouri, and Nebraska); and the *West* region includes HHS Region 8 (Colorado, Montana, North Dakota, South Dakota, Utah, and Wyoming), HHS Region 9 (Arizona, California, and Nevada), and HHS Region 10 (Alaska, Idaho, Oregon, and Washington). Data are not available for one state (Hawaii).

**Supplemental Figure 6. Weekly alcohol-related emergency department visit rates per 10,000 emergency department visits by geographic region,^a^ 2018–2020**


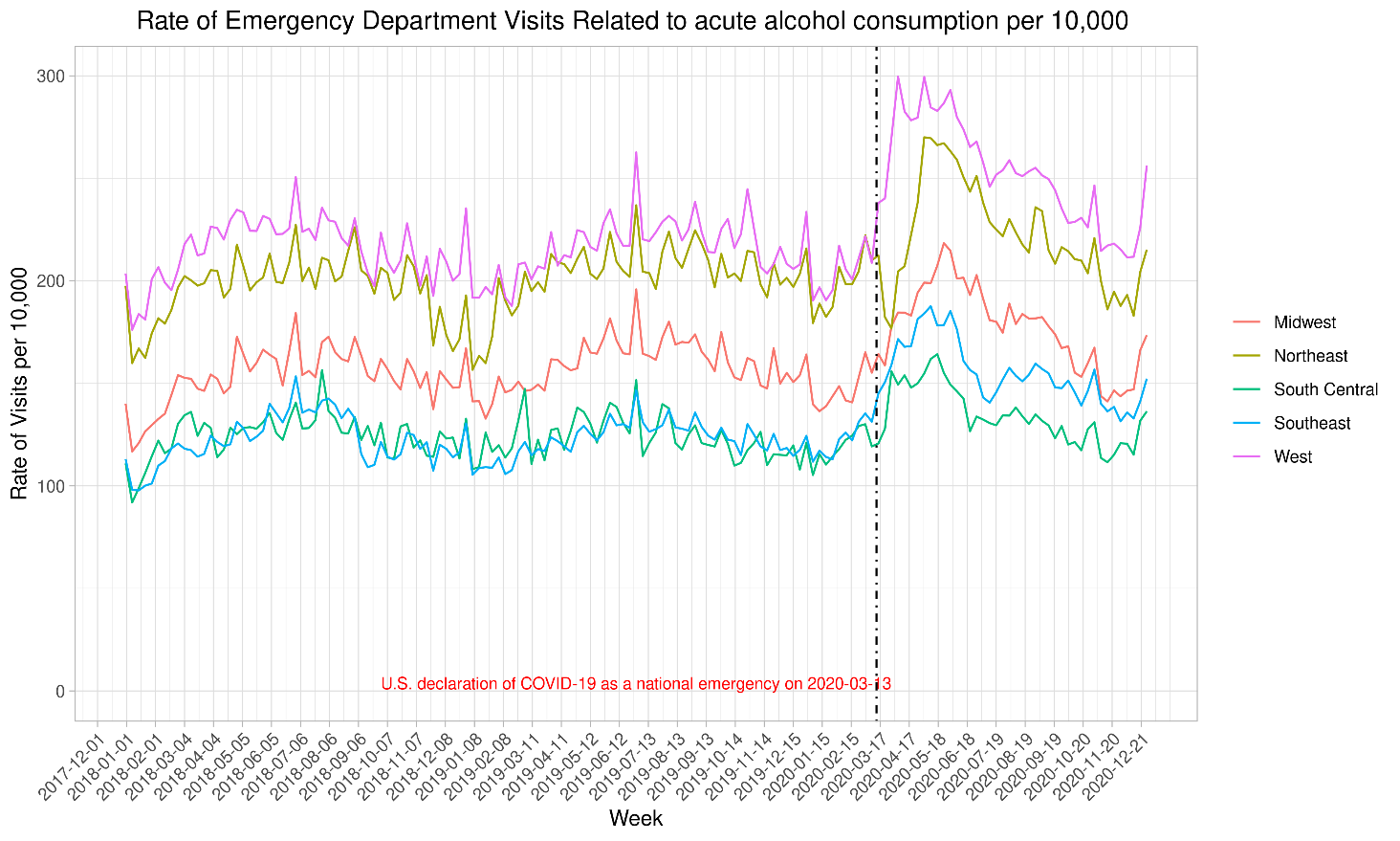


^a^ States are categorized into one of the geographic regions based on the 10 regions of the Department of Health and Human Services (HHS). The *Northeast* region includes HHS Region 1 (Connecticut, Maine, Massachusetts, New Hampshire, Rhode Island, and Vermont), HHS Region 2 (New Jersey and New York), and HHS Region 3 (Delaware, District of Columbia, Maryland, Pennsylvania, Virginia, and West Virginia); the *Southeast* region includes HHS Region 4 (Alabama, Florida, Georgia, Kentucky, Mississippi, North Carolina, South Carolina, and Tennessee); the *South Central* region includes HHS Region 6 (Arkansas, Louisiana, New Mexico, Oklahoma, and Texas); the *Midwest* region includes HHS Region 5 (Illinois, Indiana, Michigan, Minnesota, Ohio, and Wisconsin) and HHS Region 7 (Iowa, Kansas, Missouri, and Nebraska); and the *West* region includes HHS Region 8 (Colorado, Montana, North Dakota, South Dakota, Utah, and Wyoming), HHS Region 9 (Arizona, California, and Nevada), and HHS Region 10 (Alaska, Idaho, Oregon, and Washington). Data are not available for one state (Hawaii).

**Supplemental** **Figure 7. Number of weekly alcohol-related emergency department visits by the 10 regions^a^ of the Department of Health and Human Services, 2018–2020**

**
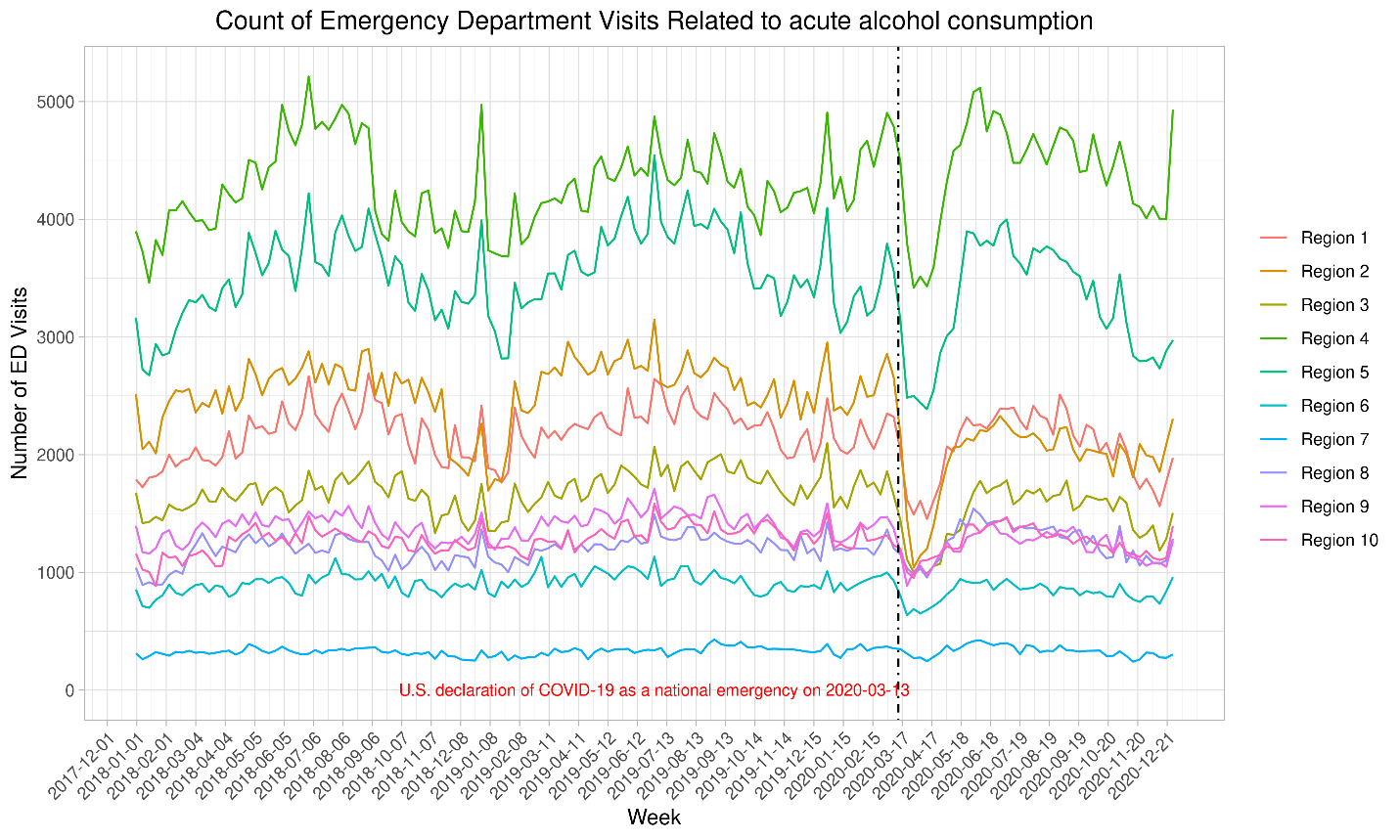
**

**^a^** States are categorized in one of the 10 regions of the Department of Health and Human Services (HHS) including Region 1: Connecticut, Maine, Massachusetts, New Hampshire, Rhode Island, and Vermont; Region 2: New Jersey and New York; Region 3: Delaware, District of Columbia, Maryland, Pennsylvania, Virginia, and West Virginia; Region 4: Alabama, Florida, Georgia, Kentucky, Mississippi, North Carolina, South Carolina, and Tennessee; Region 5: Illinois, Indiana, Michigan, Minnesota, Ohio, and Wisconsin; Region 6: Arkansas, Louisiana, New Mexico, Oklahoma, and Texas; Region 7: Iowa, Kansas, Missouri, and Nebraska; Region 8: Colorado, Montana, North Dakota, South Dakota, Utah, and Wyoming; Region 9: Arizona, California, and Nevada; Region 10: Alaska, Idaho, Oregon, and Washington. Data are not available for one state (Hawaii).

**Supplemental Figure 8. Weekly alcohol-related emergency department visit rates per 10,000 emergency department visits by HHS region,^a^ 2018–2020**


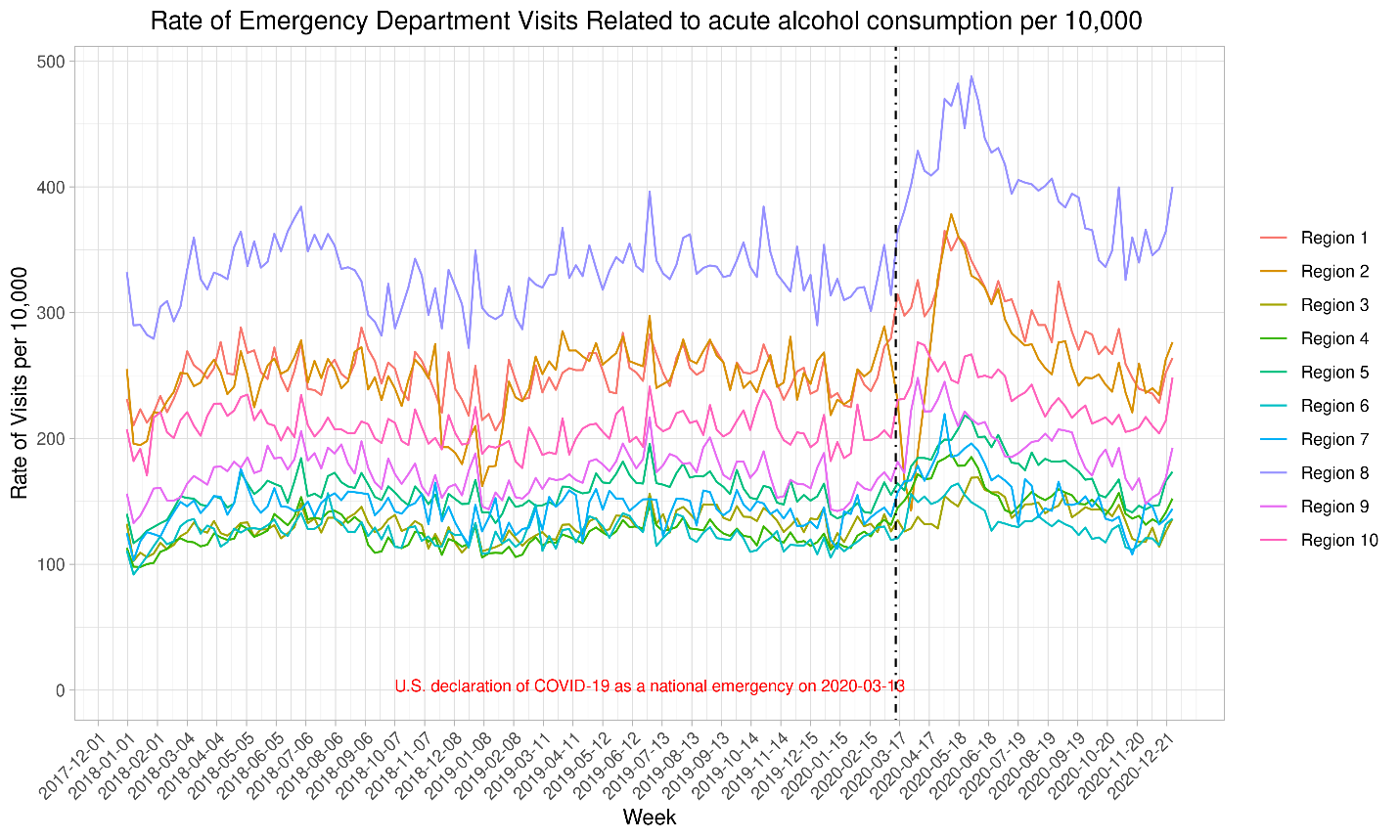


^a^ States are categorized in one of the 10 regions of the Department of Health and Human Services (HHS) including Region 1: Connecticut, Maine, Massachusetts, New Hampshire, Rhode Island, and Vermont; Region 2: New Jersey and New York; Region 3: Delaware, District of Columbia, Maryland, Pennsylvania, Virginia, and West Virginia; Region 4: Alabama, Florida, Georgia, Kentucky, Mississippi, North Carolina, South Carolina, and Tennessee; Region 5: Illinois, Indiana, Michigan, Minnesota, Ohio, and Wisconsin; Region 6: Arkansas, Louisiana, New Mexico, Oklahoma, and Texas; Region 7: Iowa, Kansas, Missouri, and Nebraska; Region 8: Colorado, Montana, North Dakota, South Dakota, Utah, and Wyoming; Region 9: Arizona, California, and Nevada; Region 10: Alaska, Idaho, Oregon, and Washington. Data are not available for one state (Hawaii).
